# Mountain-Sickness

**Published:** 1903-08-29

**Authors:** 


### Mountain-Sickness.

The summer months, even in a wet year, tempt a sufficient number of people to high altitudes to interest them in the *mal de montagne*, which, like its ally sea-sickness, is better to describe than to feel. Perhaps the best description of the condition is that given by Mr. Ed. Whymper, who investigated the subject in the Great Andes, with the object of ascertaining the heights at which the symptoms manifest themselves, and whether the effects are permanent. His first experience of mountain-sickness was at the second camp on Mount Chimborazo, at a height of 16,664 feet. He had ridden up on a mule, and says, "When we arrived we were in good condition; but in about an hour I found myself lying on my back, placed *hors de combat*, and incapable of making the least exertion. We were feverish, had intense headaches, and were unable to satisfy our desire for air, except by breathing with open mouths. This naturally parched the throat, and produced a craving for drink, which we were unable to satisfy—partly from the difficulty of obtaining it and partly from trouble in swallowing it. When we got enough we could only sip, and not to save our lives could we have taken a quarter of a pint at a draught. Before a mouthful was down we were obliged to breathe and gasp again until our throats were as dry as ever. Besides having our normal rate of breathing largely accelerated, we found it impossible to sustain life without every now and then giving spasmodic gulps just like fishes when taken out of water. Of course there was no inclination to eat; but we wished to smoke, and found that our pipes almost refused to burn, for they, like ourselves, wanted more oxygen." The condition lasted all night and all the next day, when the intensity of the symptoms diminished after repeated doses of chlorate of potash. The symptoms more usually are rapid action of the heart, vertigo, nausea, vomiting, epistaxis, hæmoptysis, headache, disturbance of the normal respiration, and a raised temperature which, in Mr. Whymper's case, was 100·4° F. There is also a disinclination to take food, and a distinct loss of muscular power, for at a little over 19,000 feet the steps become shorter and shorter, until at last the toe at one pace almost touches the heel of the other foot. There is no very satisfactory explanation of these phenomena, though experiments upon an ample scale have been made by many observers, notably by Paul Bert and by Dr. Marcet. The muscular weakness and the other symptoms are somewhat akin to that produced by bleeding, and it seems therefore as though the symptoms were dependent upon an insufficient supply of oxygen. The blood being unable to take up its normal amount from the rarefied air, there is a barometric deoxidation instead of a globular deoxidation as in bleeding. But against this theory is the fact that in balloon ascents the aeronaut may be subjected to a lower barometric pressure than any mountain climber, and yet without suffering from the symptoms of mountain-sickness. On the other hand, the climber is subjected to the low pressure for a much longer time than the aeronaut, and it is quite possible that the vaso-motor system may be competent to protect the organism for a short time, though it fails if the ordeal be too prolonged; and the suddenness with which the symptoms appear are in favour of this assumption. Those who live habitually at high altitudes obtain an immunity from mountain-sickness, for their red blood corpuscles are said to be more numerous, whilst Paul Bert calculated that the coefficient of their hæmoglobin for oxygen might rise to 17 to 21 per cent., the normal being only 10 to 12 per cent. Many remedies have been recommended for this distressing condition, most of them being directed to increasing the quantity of oxygen supplied to the body. But one of Mr. Whymper's guides declined all doctor's stuff, and contented himself with taking red wine as hot as he could make it; and the *Times* of August 21st draws attention to a somewhat similar remedy which is said to have been discovered by a Russian topographer named Passtoukhof. For some years past M. Passtoukhof has been making ascents in the Caucasus, where he has climbed the Grand Ararat, Mont Kasbek, and Mont Elbruz, the latter being the highest point in the Caucasus range and overtopping the height of Mont Blanc by more than 2,000 feet. At such an altitude as this it is easy to understand that the question of mountain-sickness becomes a serious one, and on more than one occasion M. Passtoukhof has found not only himself but all the other members of his expedition prostrated by it. On one of these occasions it occurred to him to try the experiment of lighting his spirit-lamp and making some tea, which he administered in an almost boiling condition to himself and his companions, with a result that far exceeded his expectations. Almost immediately the more serious symptoms disappeared, and in a short time all the members of the expedition found themselves well enough to continue the ascent. Later M. Passtoukhof repeated this experiment of exhibiting boiling tea as a remedy for mountain-sickness with results so invariably successful that he now feels justified in considering that it may be regarded as a specific.

